# More Than Just Cleaning: Ubiquitin-Mediated Proteolysis in Fungal Pathogenesis

**DOI:** 10.3389/fcimb.2021.774613

**Published:** 2021-11-10

**Authors:** Chengjun Cao, Chaoyang Xue

**Affiliations:** ^1^ Public Health Research Institute, Rutgers University, New Brunswick, NJ, United States; ^2^ Department of Microbiology, Biochemistry and Molecular Genetics, Rutgers University, Newark, NJ, United States; ^3^ Rutgers Center for Lipid Research, Rutgers University, New Brunswick, NJ, United States

**Keywords:** ubiquitin-proteasome system (UPS), ubiquitin, fungal pathogens, E3 ligase, drug discovery

## Abstract

Ubiquitin-proteasome mediated protein turnover is an important regulatory mechanism of cellular function in eukaryotes. Extensive studies have linked the ubiquitin-proteasome system (UPS) to human diseases, and an array of proteasome inhibitors have been successfully developed for cancer therapy. Although still an emerging field, research on UPS regulation of fungal development and virulence has been rapidly advancing and has generated considerable excitement in its potential as a target for novel drugs. In this review, we summarize UPS composition and regulatory function in pathogenic fungi, especially in stress responses, host adaption, and fungal pathogenesis. Emphasis will be given to UPS regulation of pathogenic factors that are important for fungal pathogenesis. We also discuss future potential therapeutic strategies for fungal infections based on targeting UPS pathways.

## Overview of the Ubiquitin-Proteasome System

**Figure 1:**
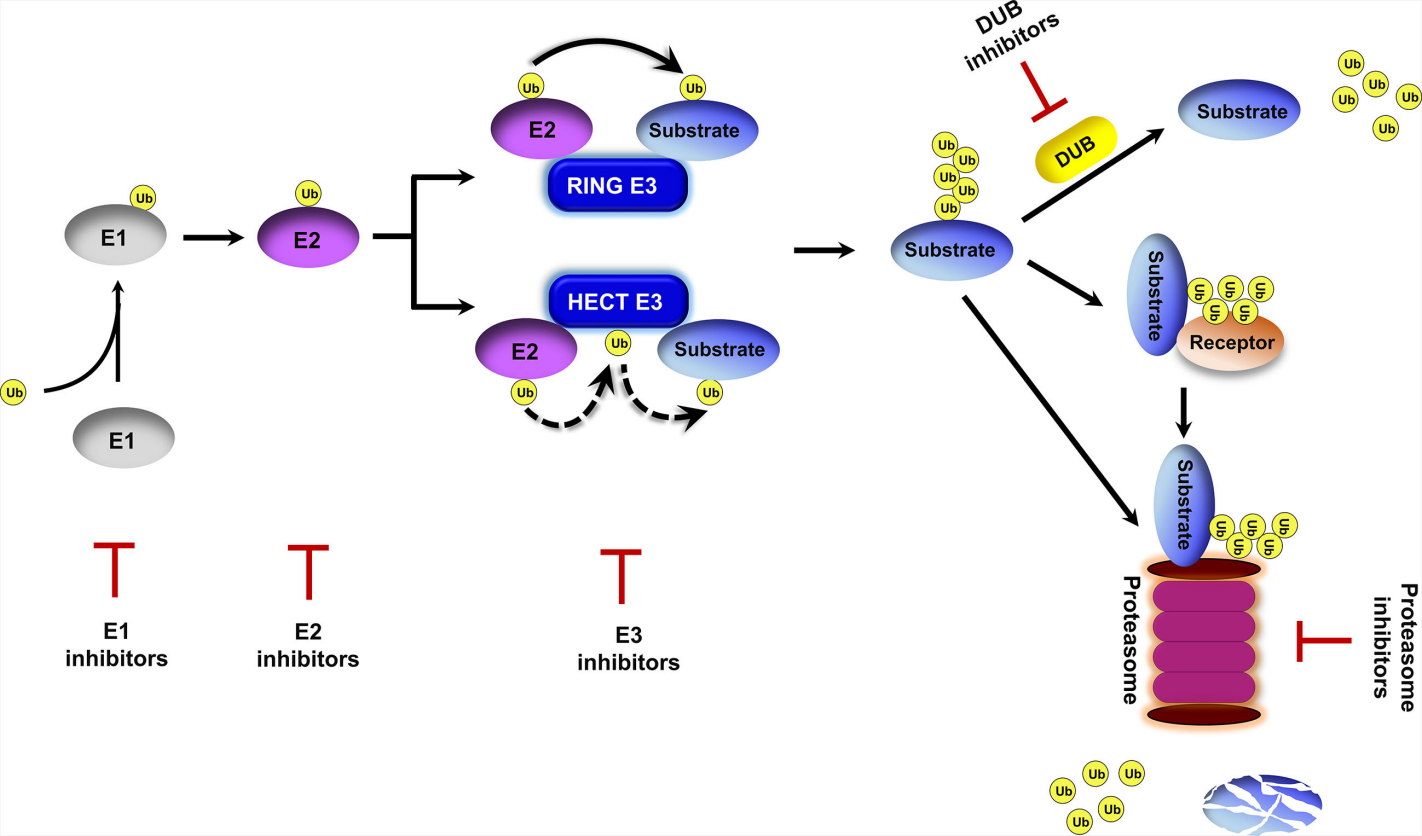
Overview of the ubiquitin proteasome system. Ubiquitin is activated by an E1 enzyme and transferred to an E2 enzyme. The activated ubiquitin is then transferred from the E2 to the substrate in a repeated process mediated by an E3 ubiquitin ligase to form a polyubiquitin chain on the protein substrate. This ubiquitination process can be reversed by deubiquitinating enzymes (DUBs). Polyubiquitinated substrates are either delivered to the proteasome by ubiquitin receptors or directly recognized by the proteasome for cleavage into small peptides and the release of ubiquitin molecules. Inhibitors targeting E1s, E2s, E3s, DUBs and the proteasome have been proposed.

Ubiquitin exists in all eukaryotic cells and it modifies proteins for proteasomal degradation and non-proteolytic functions (Finley et al., 2012). The ubiquitin-proteasome system (UPS) is important for a multitude of cellular processes due to its capability to rapidly and selectively turnover intracellular proteins. The UPS involves ubiquitin activation, ubiquitin–substrate conjugation, ubiquitin receptor recognition, and proteasomal degradation (Glickman and Ciechanover, 2002; Finley et al., 2012) (**Figure 1**). Protein ubiquitination is initiated by the ubiquitin-activating enzyme E1 in an ATP-dependent manner. Activated ubiquitin is transferred to the ubiquitin-conjugating enzyme E2. Thereafter, ubiquitin ligase E3 binds to E2 and catalyzes the covalent attachment of ubiquitin to the lysine residues of target substrates. Polyubiquitin chains are synthesized after several rounds of conjugation. Polyubiquitinated proteins are directly recognized by the 26S proteasome or delivered to the proteasome by ubiquitin receptor proteins (Elsasser and Finley, 2005; Finley et al., 2012), and then are degraded into small peptides and reusable ubiquitin (Glickman and Ciechanover, 2002). Protein ubiquitination is a reversible post-translational modification. Deubiquitinating enzymes (DUB) catalyze protein deubiquitination to prevent proteins from 26S proteasome-mediated degradation and maintain ubiquitin homeostasis in cells (Komander et al., 2009).

Given the ubiquity of the UPS that targets a wide array of proteins in various processes, it is not surprising that aberrations in the UPS have been implicated in human diseases including cancer, neurodegeneration, metabolic disorders, cardiovascular disorders, and inflammation (Glickman and Ciechanover, 2002; Zheng et al., 2016; Li et al., 2018; Senft et al., 2018; Gupta et al., 2021). Certain cancers arise from stabilization of pro-oncogenic proteins and pathways such as growth-promoting factors (Bahram et al., 2000; Yada et al., 2004; Yumimoto and Nakayama, 2020) or destabilization of tumor suppressors such as p53 and p27 (Slingerland and Pagano, 2000; Devine and Dai, 2013). The accumulation of neurotoxic proteins causes neurodegenerative diseases including Alzheimer’s disease, Parkinson’s disease, and Huntington’s disease, etc. (Popovic et al., 2014; Celebi et al., 2020). Both ubiquitination and the impairment of proteasomal function contribute to ubiquitinated protein accumulation in the cytoplasm (Zheng et al., 2016; Celebi et al., 2020). A number of compounds have been reported to target the UPS as a new class of potential therapeutics for human diseases (Yang et al., 2007; Devine and Dai, 2013; Liu et al., 2015; Hosseini et al., 2019; Wang et al., 2019a). Some proteasome inhibitors have been approved for cancer treatment, e.g., multiple myeloma and mantle cell lymphoma (Sherman and Li, 2020). The roles of various components of the UPS have been widely studied in humans and the model yeast *Saccharomyces cerevisiae*. However, studies on the role of UPS components in fungal pathogens remain limited.

Fungal diseases are serious threats to both agriculture and human health. They are difficult to treat because fungi are eukaryotic cells that share much of their cellular machinery with hosts. There are currently no vaccines in clinical use to combat fungal infections, and our armamentarium of antifungal drugs is limited compared to antibiotics and even antiviral agents (Rodrigues and Nosanchuk, 2020; Johnson, 2021). Thus, development of new treatment options is critical for controlling mycoses. The UPS tightly regulates degradation of cellular proteins that play important roles in a variety of cellular pathways during the cell life and host adaptation in fungi. Therefore, understanding the regulation of the UPS in fungal pathogenesis may be valuable for the future development of novel therapeutic approaches. Here, we will describe the role of various components of the UPS in fungal morphogenesis, stress response, host adaptation and fungal virulence.

## Ubiquitin

**Figure 2:**
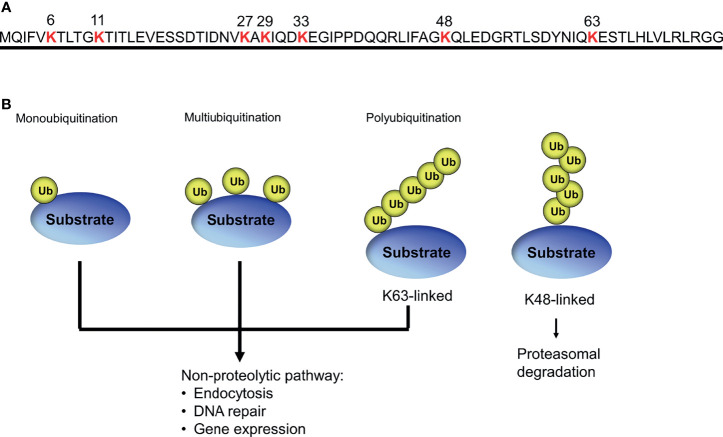
Different forms of ubiquitin modification. **(A)** Seven specific lysine residues in ubiquitin used for polyubiquitin chain formation. **(B)** Different types of ubiquitination lead to different biological outcomes.

Ubiquitin is a small polypeptide of 76 amino acids that is highly conserved in all eukaryotes (Ozkaynak et al., 1987; Hershko and Ciechanover, 1998). Ubiquitin is typically linked to protein lysine residues, as well as lysines on ubiquitin itself, allowing for different types of modification, such as monoubiquitination, multiubiquitination, and polyubiquitination (Peng et al., 2003; Komander and Rape, 2012) (**Figure 2**). The fates of ubiquitinated proteins are determined by the types of ubiquitin modification: proteins modified by K48-linked polyubiquitin chain can be preferentially degraded by the proteasome, whereas multi- or monoubiquitination often mediates non-proteolytic mechanisms such as DNA repair, protein binding, subcellular localization, and the trafficking of membrane proteins (Hicke, 2001; Kravtsova-Ivantsiv et al., 2009; Ziv et al., 2011).

In *S. cerevisiae*, ubiquitin is produced by cleavage from precursor proteins that are encoded by a family of natural gene fusions (Ozkaynak et al., 1987). *UBI1–3* genes encode hybrid proteins, in which ubiquitin units fuse to unrelated peptide sequences. *UBI4* encodes a polyubiquitin that contains five consecutive ubiquitin repeats and is highly induced under stress conditions (Finley et al., 1987; Fraser et al., 1991). In fungal pathogens, ubiquitin genes have been shown to play an important role in fungal development, stress resistance, and fungal virulence (Roig and Gozalbo, 2003; Oh et al., 2012; Chen et al., 2018). Two ubiquitin genes have been identified in *Candida albicans* (Sepulveda et al., 1996; Roig et al., 2000), an opportunistic fungal pathogen that can switch among different morphologies to adapt to various environmental stimuli (Huang, 2012). The *UBI3* gene encodes a hybrid ubiquitin fusion protein which is essential for growth of *C. albicans* (Roig and Gozalbo, 2002). The polyubiquitin gene *UBI4* contains three ubiquitin repeats and is involved in fungal growth and virulence (Sepulveda et al., 1996; Leach et al., 2011). Deletion of *UBI4* in *C. albicans* induces morphological transition from yeast to hyphae, which is important for the pathogenicity of *C. albicans* (Roig and Gozalbo, 2003; Yang D. et al., 2020). The *ubi4*∆/∆ mutant displays a cell wall biosynthesis defect when exposed to a number of cell wall stresses including treatment with the anti-fungal drug caspofungin (Leach et al., 2011). The observation that *ubi4*∆/∆ cells are more sensitive to peroxide is significant because reactive oxygen species contribute to the antimicrobial activity of host immune cells (Miller and Britigan, 1997; Leach et al., 2011). Inactivation of *UBI4* significantly attenuates virulence in a murine model of systemic candidiasis (Leach et al., 2011).

There are two ubiquitin encoding genes (*UBI1* and *UBI4*) in *Cryptococcus neoformans* (Spitzer and Spitzer, 1995), a human pathogen that commonly causes life-threatening meningoencephalitis in immunocompromised individuals (Mitchell and Perfect, 1995). *UBI1* encodes a hybrid protein that fuses with ribosomal protein Rpl40a and shares sequence similarity with *UBI1* in *S. cerevisiae*. *UBI4* encodes a polyubiquitin precursor containing five ubiquitin repeats (Spitzer and Spitzer, 1995). Deletion of *UBI1* results in a vegetative growth defect, morphological changes, a melanin production defect, decreased intracellular survival inside macrophages and virulence attenuation during infection (Zhao et al., 2020). Reconstitution of the full length of *UBI1* or the C-terminal Rpl40a could both reverse the phenotypes of *ubi1*Δ mutation, indicating a role for Ubi1 in cryptococcal ribosome biogenesis (Zhao et al., 2020). Deletion of *UBI1* also led to the differential expression of ubiquitin-conjugating enzymes (Zhao et al., 2020), suggesting a regulatory function for Ubi1 in the UPS, but the role of ubiquitin moiety in the pathogenicity of *C. neoformans* requires further investigation (Zhao et al., 2020).

Deletion of the polyubiquitin gene *MGG_01282* resulted in abnormal morphology and virulence defects in the filamentous ascomycete fungus *Magnaporthe oryzae*, the leading cause of fungal diseases in rice globally (Oh et al., 2012). Likewise, the polyubiquitin gene *CpUBI4* is required for fungal development, stress adaptation, and virulence in *Cryphonectria parasitica*, a phytopathogenic filamentous fungus that causes chestnut blight disease (Chen et al., 2018). The role of polyubiquitin genes in insect fungal pathogens were also explored recently (Wang et al., 2019b; Wang et al., 2020). Polyubiquitin genes were functionally characterized *via* gene deletion in *Metarhizium robertsii* and *Beauveria bassiana*. *MrUBI4* is involved in UV stress and the heat-shock response in *M. robertsii* (Wang et al., 2019b). Deletion of *UBI4* leads to a defect in stress tolerance and attenuated virulence in *B. bassiana* (Wang et al., 2020). In all, ubiquitin genes play important roles in fungal pathogenesis.

## Ubiquitin-Activating Enzymes (E1s)

The ubiquitination cascade is initialed by the highly conserved ubiquitin-activating enzyme (E1) that catalyzes covalent bond formation between the active site cysteine of the E1 and the C-terminal glycine residue of ubiquitin (McGrath et al., 1991; Pickart, 2001). Most species have single E1 enzyme, and its activity is necessary for all subsequent steps in ubiquitination (McGrath et al., 1991; Watts et al., 2003; Kulkarni and Smith, 2008). In *S. cerevisiae*, E1 is encoded by an essential gene *UBA1* (McGrath et al., 1991), and inactivation of *UBA1* dramatically reduces ubiquitin conjugation (Ghaboosi and Deshaies, 2007). Because of the conservative nature of the E1 in different organisms, their essentiality is likely also shared in pathogenic fungi. Therefore, despite the E1 in *S. cerevisiae* has been well characterized, there is very limited studies on its function in fungal pathogens. In the yeast pathogen *C. neoformans*, the cAMP/PKA signaling plays a critical role in capsule and melanin formation, and is important for its pathogenicity (D’Souza et al., 2001). Analysis of the PKA-regulated proteome identified the role of the ubiquitin-proteasome pathway in capsule regulation. Induction of Pka1 leads to decreased protein level of the single E1 in *C. neoformans* (Geddes et al., 2016).

## Ubiquitin-Conjugating Enzymes (E2s)

The ubiquitin-conjugating enzyme E2 is at the center of the E1–E2–E3 enzymatic cascade. E2s interact with E1 and E3 and transfer ubiquitin from E1 to the substrate (Ye and Rape, 2009). E2s belong to the E2 superfamily which contains a highly conserved ubiquitin-conjugating (UBC) domain (Ye and Rape, 2009). A total of 13 E2 genes have been found in *S. cerevisiae* (Finley et al., 2012), 40 in *A. thaliana* (Kraft et al., 2005), and 37 in humans (van Wijk and Timmers, 2010). E2 enzymes have the ability to control the topology of ubiquitin conjugates and to determine the fate of labeled proteins to either degradation or nonproteolytic processes (van Wijk and Timmers, 2010). Multiple E2 enzymes can be involved in ubiquitination of a single substrate, such as the Matα2 transcriptional regulator in *S. cerevisiae* that is controlled by 4 E2s (Chen et al., 1993). A single E2 can combine with different types of E3s that recognize and select distinct substrates (Finley et al., 2012). Interestingly, E2s have also been shown to directly ubiquitinate endogenous substrates independent of an E3 ligase (Berleth and Pickart, 1996; Kraft et al., 2005). Several studies on E2 enzymes in plants have revealed the role of E2 enzymes in host defense against fungal infection (Finley et al., 2012; Liu et al., 2020). Plant E2s are involved in abiotic stress responses, including osmotic stress tolerance (Zhou et al., 2010; Chung et al., 2013), heat shock responses (Feussner et al., 1997), and oxidative stress responses (Zhou et al., 2010). E2 enzymes also play important roles in plant immune responses and DNA repair (Finley et al., 2012; Liu et al., 2020). Inactivation of the ubiquitin-conjugating enzyme 4 (Tau4) in bread wheat *Triticum aestivum* increases host defense against the phytopathogen *Zymoseptoria tritici* (Millyard et al., 2016). Ectopic expression of the E2 gene *OgUBC1* from rice confers resistance against *Botrytis cinerea* infection in *A. thaliana* (Jeon et al., 2012). The ubiquitin-conjugating enzyme Rad6 is conserved amongst eukaryotes (Huang et al., 1997; Worthylake et al., 1998). *Rad6* in *C. albicans* protects the fungus against UV damage and negatively regulates hyphal development (Leng et al., 2000). MoRad6 in *M. oryzae* is essential for fungal development and pathogenicity (Shi et al., 2016). There is no published work on UBC domain containing proteins in *C. neoformans* or *Aspergillus fumigatus*. Sequence homology searching revealed 18 proteins containing the UBC domain in *C. neoformans* genome (https://fungidb.org).

## Ubiquitin Ligases (E3s)

Ubiquitin ligases (E3s) form a large and diverse family that recognize specific substrate proteins. The E3 ligases bind E2s and substrates to facilitate substrate-specific ubiquitination (Hicke and Dunn, 2003; Finley et al., 2012). E3s are classified into two major classes, which catalyze ubiquitin transfer from E2 to substrate *via* different mechanisms. These two classes are either RING (really interesting new genes) domain-containing E3s, or HECT (homologous to E6-AP C-terminus) domain E3s (Huibregtse et al., 1995; Bernassola et al., 2008). HECT E3s form an intermediate thioester bond between the active site cysteine in the HECT domain and ubiquitin received from E2 and transfer ubiquitin to substrate from the HECT E3-Ub (Huibregtse et al., 1995). RING E3s catalyze the direct transfer of ubiquitin from the E2 to substrates by bridging the interaction between E2 and substrate proteins (Deshaies and Joazeiro, 2009). In addition, RBR proteins (RING-between-RINGs domain E3, a subclass of RING domain E3s) bind E2s with one RING domain and transfer ubiquitin to the other RING domain before its transfer to the substrate, therefore apparently functioning as RING/HECT hybrids (Wenzel et al., 2011; Wenzel and Klevit, 2012). There are only five HECT domain E3s among 60–100 putative E3s in yeast (Finley et al., 2012) that function in diverse processes ranging from protein degradation, metabolic process, endocytosis, and cell cycle progression (Pesin and Orr-Weaver, 2008; Rotin and Kumar, 2009; Finley et al., 2012). Most identified E3s belong to the class of RING domain E3s. Cullin-RING ligases (CRLs) are the largest group of ubiquitin ligases in eukaryotes (Finley et al., 2012). The SCF (Skp1-Cullin-F-box protein) ligase is the typical CRL ligase, in which the cullin protein Cdc53 binds to the small RING domain subunit in Rbx1 with its C-terminal regions and binds to F-box proteins through the linker protein Skp1 with the N- terminal regions (Finley et al., 2012). Substrates of SCF ligases are phosphorylated to create a binding surface for F-box motif recognition (Feldman et al., 1997; Skowyra et al., 1997).

The role of E3 ligases has been widely studied in human health and disease, including cancer, neurodegenerative disease, and neurological syndromes, with a view to develop new clinical therapies (Huang et al., 2016; Mao et al., 2018; Pan, 2020; Wang Y. et al., 2020; Goto et al., 2021). Studies on E3s in pathogenic fungi have revealed that many E3s are required for fungal virulence, as reviewed by (Liu and Xue, 2011). These studies focus on the homologs of Grr1 and Cdc4, which are the two best studied SCF ligases in *S. cerevisiae*. SCF(Grr1) induces degradation of the Mth1 and G1 cyclins Cln1 and Cln2 to control glucose sensing and the cell cycle in *S. cerevisiae* (Seol et al., 1999; Skowyra et al., 1999; Flick et al., 2003). SCF(Cdc4) controls cell cycle transitions and nutrient responses by recruiting downstream phosphorylated substrates, such as the cyclin-dependent kinase inhibitor Sic1 and the transcription factor Gcn4 (Feldman et al., 1997; Skowyra et al., 1997; Meimoun et al., 2000; Chi et al., 2001). The role of other major subunit of SCF complex, such as cullin Cdc53, has also been reported in pathogenic fungi (Trunk et al., 2009; Sela et al., 2012). Cdc53 is an essential gene in *C. albicans* and negatively regulates filamentation when under the control of a tetracycline-dependent promoter (Trunk et al., 2009). Modification of Cdc53 by the ubiquitin-related protein NEDD8/Rub1 (neddylation, ubiquitin-related protein) is also involved in morphogenetic phenotype (Sela et al., 2012). Either deletion of *CaRUB1* or mutation of the neddylation target site in CaCdc53 showed filamentous growth, indicating that CaCdc53 neddylation regulates *Candida* dimorphic switch (Sela et al., 2012).

Grr1 contains an F-box domain and a leucine rich repeat (LRR) at its C-terminus. Homologs of Grr1 have been reported in several important fungal pathogens (Liu and Xue, 2011). Fbp1 is essential for *Cryptococcus* virulence. Even though the *fbp1*∆ mutant produces normal classical virulence factors, including capsule and melanin production, mice infected with *fbp1*∆ showed a low persistence of fungal burden throughout infection, (Liu et al., 2011; Liu and Xue, 2014). Further studies found that *fbp1*Δ-infected mice developed a robust Th1 and Th17 host protective immune response that helps contain yeast cells in the lung and prevent them from dissemination to maintain the long-term survival of the host (Masso-Silva et al., 2018). Mice challenged with heat-killed *fbp1*Δ cells (HK-*fbp1*∆) also can develop a robust Th1 response that confers protection against infection with the virulent wild type strain (Masso-Silva et al., 2018). Interestingly, vaccination of HK-*fbp1*Δ cells induces cross-protection against challenge with other invasive fungal pathogens, including *C. gattii* and *A. fumigatus*. The vaccine protection is effective even in mice depleted of CD4^+^ T cells, a condition mimics HIV/AIDS-induced immune deficiency (Wang Y. et al., 2019). Several potential substrates of Fbp1 have been identified in *C. neoformans* (Liu et al., 2011; Liu and Xue, 2014; Han et al., 2020; Fan and Liu, 2021). One of them, a Cdk-related kinase Crk1, has been found to be involved in meiosis regulation (Liu et al., 2011; Liu and Shen, 2011). Another substrate Isc1 (the inositol phosphosphingolipid-phospholipase C1) is required for fungal survival inside macrophage cells (Liu and Xue, 2014). Several additional Fbp1 interacting proteins, including mannoprotein Cmp1 and vacuolar morphogenesis protein Vlp1, has also been considered as potential substrates of Fbp1 based on protein pull-down assays, although their functional verification remains missing. Both proteins are required for pathogenicity of *C. neoformans*, as the *vlp1*Δ mutant is avirulent and the *cmp1*Δ mutant or the *CMP1* overexpression strain shows virulence attenuation in a murine infection model of systemic cryptococcosis (Han et al., 2020; Fan and Liu, 2021). Besides Fbp1, there are 19 additional F-box proteins in *C. neoformans* that remain to be studied.

In *C. albicans*, Grr1 negatively regulates pseudohyphal development (Butler et al., 2006), an important fungal virulence factor implicated in the cell cycle (Bachewich and Whiteway, 2005; Bensen et al., 2005; Chapa y Lazo et al., 2005; Li et al., 2006). *A. fumigatus* GrrA shares structural similarity to Fbp1 of *C. neoformans*. Deletion of *GRRA* in *A. fumigatus* has no impact on its virulence, indicating the role of Grr1 homologs in virulence might vary in different pathogenic fungi (Johnk et al., 2016). In *Gibberella zeae*, an important plant pathogen and fungal mycotoxins producer, the Grr1 homolog Fbp1 is a versatile F-box protein involved in the mycelial growth, sexual reproduction, and virulence. Deletion of *FBP1* impairs the ability of *G. zeae* to colonize plant cells, which leads to attenuated virulence with mild disease (Han et al., 2007). Two homologs of Grr1 have been identified in *M. oryzae*. Both of them are required for fungal pathogenicity based on mutagenesis studies (Sweigard et al., 1998; Guo et al., 2015).

Cdc4 homologs have also been well characterized in a number of fungal pathogens. Cdc4 contains an F-box domain and a WD40 domain that is important for interaction with its downstream substrates. SCF(Cdc4) plays critical roles in *C. albicans* morphogenesis and biofilm formation (Atir-Lande et al., 2005; Shieh et al., 2005; Chin et al., 2013; Tseng et al., 2015). Deletion of *CDC4* promotes hyphae growth, especially of true hyphae rather than pseudohyphal development, which is different from Grr1 in this fungus. Hyphae growth in the *cdc4*∆/∆ mutant is independent of the transcription factors Efg1 and Cph1 or G1 cyclins (Atir-Lande et al., 2005). Multiple Cdc4 substrates important for Cdc4-mediated morphogenesis have been identified. Sol1, a homologue of Sic1 (a substrate of Cdc4 in *S. cerevisiae*), has been identified as a substrate of Cdc4 in *C. albicans* and it involved in *C. albicans* morphogenesis. Another SCF(Cdc4) substrate, Ume6, has been found to be critical target of SCF(Cdc4) and responsible for the hyphae formation of *cdc4*∆/∆ mutant in *C. albicans*. The *cdc4*∆/∆*ume6*∆/∆ double mutant has reduced filamentous growth, but more pseudohyphal development than the *cdc4*∆/∆ single mutant (Mendelsohn et al., 2017). Consistent with this observation, deletion of *UME6* blocks the increased expression of hypha-specific genes in the *cdc4*∆/∆ mutant background. The *cdc4*∆/∆*ume6*∆/∆*sol1*∆/∆ triple mutant lost the ability for filamentous growth, while can be restored by introducing of *UME6*, indicating the important role of Ume6 in Cdc4-mediated hyphal formation (Mendelsohn et al., 2017). Thr1 has also been identified as a Cdc4 substrate by *in vitro* affinity purification in *C. albicans* (Tseng et al., 2010). Thr1 is a homoserine kinase and its stability is dependent on Cdc4. The *thr1*∆/∆ mutant accumulates toxic homoserine and shows virulence attenuation (Kingsbury and McCusker, 2010a; Kingsbury and McCusker, 2010b). Similar to Sol1, Thr1 positively modulates hyphal formation (Lee et al., 2018). In addition, Thr1 negatively regulates biofilm formation in *C. albicans* (Tseng et al., 2015).

There are over 20 proteins containing the F-box domain in the genomes of *Aspergillus* spp. e.g., human pathogen *A. fumigatus* and mycotoxin producing plant pathogen *A. flavus* (Frawley and Bayram, 2020). A total of 26 F-box proteins in *A. fumigatus* and 30 in *A. flavus* have been detected by immunoprecipitation of the HA-tagged SkpA adaptor protein during vegetative growth. Analysis of fungal F-box proteins has revealed an interaction network in the presence of various exogenous stress conditions, including osmotic and oxidative stresses, the cell wall stressor Congo red, and the antifungal drug Amphotericin B or Miconazole (Frawley and Bayram, 2020). While some F-box proteins were identified to interact with SkpA across all these different stress conditions, other F-box proteins were only detected under one condition, suggesting they may be specific to one particular stress. Two F-box proteins (Fbx20 and Fbx45) in *A. fumigatus* and three F-box proteins (Fbx1, Fbx11 and Fbx45) in *A. flavus* interact with SkpA only in response to Amphotericin B. Fbx45 was detected in both species in response to Amphotericin B. Orthologs of Fbx45 are conserved among *Aspergillus* species and do not exist in humans, suggesting that Fbx45 could be a potential anti-fungal target (Frawley and Bayram, 2020). Fbx15 is specific to filamentous fungi. Deletion of the F-box domain in Fbx15 impairs stress resistance, similar to the *fbx15*Δ mutant, suggesting that Fbx15 functions as a subunit of the SCF(Fbx15) E3 ligase and is critical for stress tolerance in *A. fumigatus*. The *fbx15*Δ mutant cells are cleared at an early stage of infection and the infected animals show no disease symptom in a mouse model of aspergillosis, indicating that Fbx15 is required for *Aspergillus* virulence (Johnk et al., 2016).

Other F-box proteins, such as Frp1 in the root-infecting fungus *Fusarium oxysporum* and several F-box proteins in *M. oryzae*, including MoFwd1, MoCdc4 and MoFbx15, and HECT type E3 Upl3 have also been reported to be required for fungal virulence (Duyvesteijn et al., 2005; Jonkers et al., 2011; Shi et al., 2019; Li et al., 2020).

Overall, because the F-box protein family has large members with diverse functions and the fact that the F-box protein determines the substrate specificity, much of attention on the E3 ligase studies in fungal pathogens have been focusing on function of F-box proteins and their substrates. Understanding the function of F-box proteins and their substrates will lead to a better understanding of the UPS mediated functional regulation, including fungal development and pathogenesis. Identification of F-box proteins that are important for fungal virulence may also lead to development of novel drug targets, hence is highly significant.

## Deubiquitinating Enzymes (DUBs)

Deubiquitinating enzymes (DUBs) reverse ubiquitination by hydrolyzing polyubiquitin chains (Amerik and Hochstrasser, 2004; Reyes-Turcu et al., 2009). DUBs are an important part of the UPS in multiple fundamental cellular processes, including DNA damage repair, protein quality control and cell cycle regulation (Komander, 2009; He et al., 2016; Kee and Huang, 2016). DUBs have been associated with human pathologies including cancer, neurodegenerative diseases and infectious diseases (Nanduri et al., 2013; Heideker and Wertz, 2015; He et al., 2016; Das et al., 2020). DUBs are involved in the regulation of various stress responses in *S. cerevisiae* (Auesukaree et al., 2009; Mat Nanyan et al., 2020) and *C. neoformans* (Liu et al., 2008; Fang et al., 2012). There are 19 putative DUB proteins in *C. neoformans* and most of them are required for its pathogenesis (Liu et al., 2008; Fang et al., 2012). Deletion of *UBP5* elevates sensitivity to external stress such as high temperature, reactive oxygen species, osmotic changes, or antifungal agents. Ubp5 and Doa4 are important for *Cryptococcus* virulence factor production (capsule and melanin) and *in vivo* fungal virulence (Fang et al., 2012). Several studies have identified the role of DUBs in the pathogenic fungus *M. oryzae*. Deletion of *MoUBP14* resulted in virulence attenuation and phenotypic defects, including stress sensitivity and reduced conidiation (Wang et al., 2018). MoUbp4 and MoUbp8 are required for infection-related morphogenesis and pathogenicity. The *moubp4*Δ and *moubp8*Δ mutants show reduced mycelial growth, which blocks penetration and invasive growth and reduces pathogenicity (Que et al., 2020; Yang J. et al., 2020). Overall, the deubiquitination process provides another layer of post-translation regulation that has been shown to be important for fungal pathogenesis.

## Ubiquitin Receptors

How do proteasomes specifically acquire ubiquitinated proteins? Studies in *S. cerevisiae* have revealed that several ubiquitin receptors, including Rad23, Ddi1, and Dsk2, play important roles in shuttling substrates to the proteasome (Verma et al., 2004; Dantuma et al., 2009). These three ubiquitin receptors contain both ubiquitin-like (UBL) domains and ubiquitin-associated (UBA) domains. Ubiquitin receptors specifically bind to polyubiquitin chains of target proteins through UBA domains and interact with the proteasome through their UBL domain (Dantuma et al., 2009). In yeast, Rad23 and Ddi1 are two DNA damage-inducible proteins (Liu et al., 1997; Jelinsky et al., 2000) and Dsk2 is required for the duplication of microtubule-organizing centers (Biggins et al., 1996). Rad23 contributes to both nucleotide excision repair (NER) and protein turnover by directing proteins to the proteasome from yeast to human (Dantuma et al., 2009; Farmer et al., 2010; Lahari et al., 2017). Rad23 has a UBL domain and two UBA domains (UBA1 and UBA2). Its UBA2 domain binds to ubiquitin molecules and the UBL domain binds to proteasome (Dantuma et al., 2009). In addition, it also contains a XPCB (Rad4-binding) domain that is important for NER. In *C. albicans*, either deletion or overexpression of *RAD23* leads to increased UV sensitivity. The *rad23*∆/∆ mutant showed hypervirulence in a murine infection model (Feng et al., 2020). Rad4 is critical in the Rad23 mediated response to UV and has similar functions regulating cell morphogenesis and biofilm formation, yet it does not play an important role in fungal virulence. Since Rad4 is a key component of the NER pathway and is disposable for fungal virulence, Rad23 mediated virulence suppression is likely due to its role in protein degradation, rather than the NER pathway in *C. albicans* (Feng et al., 2020). Deletion of different domains to uncouple the two functions of Rad23 has also been reported in *C. neoformans*. In the wax moth (*Galleria mellonella*) larvae infection model, the UBA2 domain is required for virulence and the XPCB domain is not involved in fungal virulence in *C. neoformans*, indicating that the role of Rad23 in virulence is also due to its function in protein turnover rather than NER. How the ubiquitin receptor Rad23 regulates virulence remains to be understood (Verma et al., 2019).

## The Proteasome

The proteasome is found in all eukaryotes and has been extensively characterized in *S. cerevisiae* (Finley et al., 2012). The proteasome is a highly conserved protein complex composed of a catalytic 20S core particle and associated 19S regulatory particles. The regulatory particles recognize ubiquitin–protein conjugates or shuttle receptors with their cargo of ubiquitinated substrates, deubiquitinate substrates, and translocate proteins into the core particle for hydrolysis. The core particle contains interior proteolytic active sites that control proteolysis (Finley, 2009; Finley et al., 2012; Enenkel, 2014). Although the proteasome has not been extensively characterized in pathogenic fungi, it has been implicated in fungal virulence (Geddes et al., 2016; Hossain et al., 2020). Proteasome inhibitors targeting the 20S subunit of the proteasome were used to identify the role of the proteasome in fungal virulence. Commonly used proteasome inhibitors are bortezomib, a boronic acid-based inhibitor widely used for the treatment of multiple myeloma and hematologic malignancy (Nunes and Annunziata, 2017), and MG132, a synthesized peptide aldehyde commonly used for *in vitro* studies (Lee and Goldberg, 1998).

Among the 33 predicted proteasome subunit genes in *C. albicans*, 28 are essential subunits. Genetic depletion of most proteasome subunits induces filamentation, demonstrating that proteasome function is essential for *C. albicans* growth and morphogenesis (Hossain et al., 2020). Proteasome inhibitor treatment also produces filaments that share structural similarities with the *hsp90*∆/∆ mutant in *C. albicans*. Inhibition of the proteasome relieves Hsp90-mediated repression of cAMP-PKA signaling to induce morphogenesis (Hossain et al., 2020). To assess the role of the proteasome in the filamentation of other *Candida* species, including *C. dubliniensis*, *C. tropicalis*, *C. krusei*, *C. parapsilosis*, and *C. auris*, Bortezomib has been used to treat these fungi. Bortezomib treatment results indicate that the proteasome has conserved roles in regulating fungal morphogenesis across diverse fungal species (Hossain et al., 2020). Bortezomib treatment impairs the growth of *C. neoformans* strains and reduces capsule size and cell size, suggesting that the proteasome function is involved in virulence factor production in *C. neoformans* (Geddes et al., 2016). Because of the conservative nature of the proteasome function in eukaryotes, there is a legitimate concern about the toxicity of utilizing proteasome inhibitors as potential antifungal drugs. Once identified a potent inhibitor, employing medicinal chemistry to further modify the compound to improve its specificity may increase the feasibility for its further development.

## UPS-Based Drug Discovery

With improved understanding of the UPS in regulating protein function and its role in human diseases, it has been realized that inhibitors targeting specific components of the UPS may have significant therapeutic potential (Veggiani et al., 2019). The clinical importance of such components has been demonstrated by the success of the proteasome inhibitors Bortezomib (PS341/Velcade, approved by the US Food and Drug Administration (FDA) in 2003) and Carfilzomib (PR-171/Kyprolis, approved by the FDA in 2012) in anti-cancer therapy (Richardson et al., 2005; Rentsch et al., 2013). Given the essential role of E1s in globally controlling protein ubiquitination in cells, inhibition of E1s is predicted to be non-specific (Zhang and Sidhu, 2014). It could be feasible to develop E2 inhibitors that selectively target E2-E3 interaction interfaces, with better specificity than targeting the interfaces of E1–E2 and E2–Ub (Zhang and Sidhu, 2014). E3s determine specific substrates for ubiquitination and thus enable selective targeting of a limited number of proteins. Inhibition of E3s may increase the effectiveness of treatment with fewer side effects compared to general UPS inhibitors. Several small molecule inhibitors of E3 ligases have been reported (Zhang and Sidhu, 2014). MDM2 is a RING E3 ligase that regulates the abundance of the tumor suppressor p53 (Reifenberger et al., 1993). Small molecules such as Nutlins and RITA, disrupt the interaction between MDM2 and p53 to mediate p53 ubiquitination and have significant anti‐tumor effects (Issaeva et al., 2004). Inhibitors of several SCF family members have also been identified, e.g., CpdA prevents the incorporation of Skp2 into the SCF^skp2^ complex (Chen et al., 2008), while SCF‐I2 and SMER3 inhibit Cdc4 and Met30 (Aghajan et al., 2010), respectively. Significant progress has been made to identify inhibitors targeting DUBs because of the substrate specificity and well-defined catalytic pockets in DUBs (Zhang and Sidhu, 2014). Therefore, it is likely feasible to identify small molecules that selectively target specific UPS components.

Several emerging technologies have been developed to manipulate the UPS, aiding drug discovery in this domain (Veggiani et al., 2019). PROTAC (proteolysis-targeting chimeras) technology represents a promising strategy for drug discovery in cancer research (Sakamoto et al., 2001). PROTACs are hetero-bifunctional molecules that recruit an E3 for precise degradation of drug targets (Toure and Crews, 2016), therefore controlling target specificity and reducing systemic toxicity *in vivo* (Bondeson et al., 2015). Some PROTACs, such as ARV-110 (an oral protein degradation agent) and ARV-471 (an estrogen receptor (ER) alpha PROTAC molecule), have shown encouraging results in clinical trials (Qi et al., 2021). In yeast, a ligase-trapping system has been developed to aid the identification of E3 ligase targets (Mark et al., 2014; Mark et al., 2016). Although it is not directly utilized for drug discovery, this system will aid the identification of novel E3 substrates, which could be valuable for future drug target discovery. In this system, the E3 is fused with the UBA domain from the ubiquitin receptor proteins Rad23 or Dsk2. The polyubiquitin-binding domain in the E3 ligase increases the binding affinity to its ubiquitinated substrates, allowing identification of weak and transit substrate proteins. In addition, the commercialization of antibodies that recognize the Lys-ϵ-Gly-Gly (K-ϵ-GG) remnant, which is produced by trypsin digestion of proteins containing ubiquitinated lysine residues, improved the ability to analyze the ubiquitinated substrates and identify ubiquitination sites (Udeshi et al., 2013). Although still understudied and no proven inhibitor against fungal UPS in clinical trial, technologies developed in other systems should provide powerful tools for UPS research in fungal pathogens, and may aid the future drug discovery targeting UPS in fungi.

## Concluding Remarks

Invasive fungal infections are serious threats to human health with estimated 1.5 million deaths each year (Brown et al., 2012). They are difficult to treat because fungi are eukaryotic cells that share much of their cellular machinery with hosts. The close evolutionary relationship between human and fungi hampers the development of antifungal drugs (Robbins et al., 2016). There is a clear medical need to develop new antifungal drugs. Given that the UPS plays pivotal cellular roles in diverse fungal pathogens and has a track record as a proven drug target in anti-cancer drug development, selective targeting of UPS components provides a promising therapeutic strategy to mitigate antifungal resistance and combat fungal infections.

Studies on UPS components in fungal pathogens remains limited and further studies are warranted to better understand the regulation of UPS in fungal development and virulence. Drug development against targets in the fungal UPS has not been explored extensively. Distinguishing UPS proteins in pathogenic fungi from their hosts and selective targeting of the fungal UPS and its fungal function-specific substrates will be crucial steps to develop a UPS inhibitor into a potential antifungal drug. Improved understanding of the specificity of E3s and their downstream substrates in the fungal UPS will increase the feasibility of targeting the UPS as a promising antifungal strategy. UPS substrates that are involved in some unique processes in fungal pathogens, such as cell wall regeneration, morphologic changes, spore germination and certain virulence factors development, are potential antifungal targets. Therefore, research on the fungal UPS system will not only improve our understanding of the molecular basis of UPS regulation of fungal development and pathogenesis, but also may lead to a valuable new avenue of antifungal drug discovery.

## Author Contributions

CC and CX designed the review, wrote and edited the manuscript. All authors reviewed and approved the final manuscript. All authors contributed to the article and approved the submitted version.

## Funding

This work was supported by the NIH grants R01AI141368, R01AI123315, and R21AI154318 to CX.

## Conflict of Interest

The authors declare that the research was conducted in the absence of any commercial or financial relationships that could be construed as a potential conflict of interest.

## Publisher’s Note

All claims expressed in this article are solely those of the authors and do not necessarily represent those of their affiliated organizations, or those of the publisher, the editors and the reviewers. Any product that may be evaluated in this article, or claim that may be made by its manufacturer, is not guaranteed or endorsed by the publisher.
